# Keloid Biomarkers and Their Correlation With Immune Infiltration

**DOI:** 10.3389/fgene.2022.784073

**Published:** 2022-06-02

**Authors:** Xufeng Yin, Wenbo Bu, Fang Fang, Kehui Ren, Bingrong Zhou

**Affiliations:** ^1^ Department of Dermatology, The First Affiliated Hospital of Nanjing Medical University, Nanjing, China; ^2^ Department of Dermatologic Surgery, Dermatology Hospital of Chinese Academy of Medical Sciences, Nanjing, China

**Keywords:** keloid, biomarker, gene expression, pathogenesis, immune infiltration

## Abstract

**Objective:** This work aimed to verify the candidate biomarkers for keloid disorder (KD), and analyze the role of immune cell infiltration (ICI) in the pathology of keloid disorder.

**Methods:** The keloid-related datasets (GSE44270 and GSE145725) were retrieved from the Gene Expression Omnibus (GEO). Then, differential expressed genes (DEGs) were identified by using the “limma” R package. Support vector machine-recursive feature elimination (SVM-RFE) and LASSO logistic regression were utilized for screening candidate biomarkers of KD. The receiver operating characteristic (ROC) curve was used to evaluate the diagnostic power of candidate biomarkers. The candidate biomarkers were further verified through qRT-PCR of keloid lesions and the matched healthy skin tissue collected from eight cases. In addition, ICI in keloid lesions was estimated through single-sample gene-set enrichment analysis (ssGSEA). Finally, the potential drugs to the treatment of KD were predicted in the Connectivity Map Database (CMAP).

**Results:** A total of 406 DEGs were identified between keloid lesion and healthy skin samples. Among them, STC2 (AUC = 0.919), SDC4 (AUC = 0.970), DAAM1 (AUC = 0.966), and NOX4 (AUC = 0.949) were identified as potential biomarkers through the SVM-RFE, LASSO analysis and ROC analysis. The differential expressions of SDC4, DAAM1, and NOX4 were further verified in collected eight samples by qRT-PCR experiment. ICI analysis result showed a positive correlation of DAAM1 expression with monocytes and mast cells, SDC4 with effector memory CD4^+^ T cells, STC2 with T follicular helper cells, and NOX4 with central memory CD8^+^ T cells. Finally, a total of 13 candidate small molecule drugs were predicted for keloids treatment in CMAP drug database.

**Conclusion:** We identified four genes that may serve as potential biomarkers for KD development and revealed that ICI might play a critical role in the pathogenesis of KD.

## Introduction

Keloid disorder (KD) can be triggered by skin trauma, such as infection, insect bites, burn, surgery, and other injuries (Gauglitz et al., 2011; Lawrence et al., 2012). KD is often accompanied with unbearable pruritus and pain (De Felice et al., 2007). Current treatments for KD mainly include laser and 5-fluorouracil injections, intralesional steroids injection, surgery, cryotherapy, pressure therapy, radiotherapy, and silicone. However, satisfactory outcomes are hard to achieve in clinical practice (Gauglitz, 2013).

KD etiology remains largely unclear, probably involving skin tension (Song et al., 2019), tissue hypoxia (Wolfram et al., 2009), chronic inflammation (Ogawa, 2017), autoimmunity (Kazeem, 1988), and genetics factors (Bayat et al., 2005). The pathogenesis of KD at the molecular level can be explored by *in-vitro* cell culture models using high-throughput technology (Broek et al., 2014). The Gene Expression Omnibus (GEO) database established by the National Center for Biotechnology Information (NCBI) in 2000 provides gene expression profiles obtained from high-throughput technique. Bioinformatic techniques can be used to identify biomarkers for KD development, thus providing a possibility to design new diagnostic tools for KD.

Immune cell infiltration (ICI) contributes to keloid genesis and progression (Kazeem, 1988; Jiao et al., 2015). For instance, genes related to M2 macrophages and Foxp3-expressing regulatory T cells are up-regulated in keloid lesions (Jin et al., 2018). Chen et al. found that the overproduction of collagen was related to the abnormal level of regulatory T cells within keloid lesions Chen et al. (2019). This inspires us to delve into KD pathogenesis through the analysis of ICI.

In the present study, we comprehensively examined GEO database-derived data using differential gene expression analysis, enrichment analysis, PPI network analysis. The bioinformatic findings were validated with experiments. Besides, this study also examined the association of keloid biomarkers with infiltrating immune cells.

## Materials and Methods

### Data Collection and Quality Control

**FIGURE 1:**
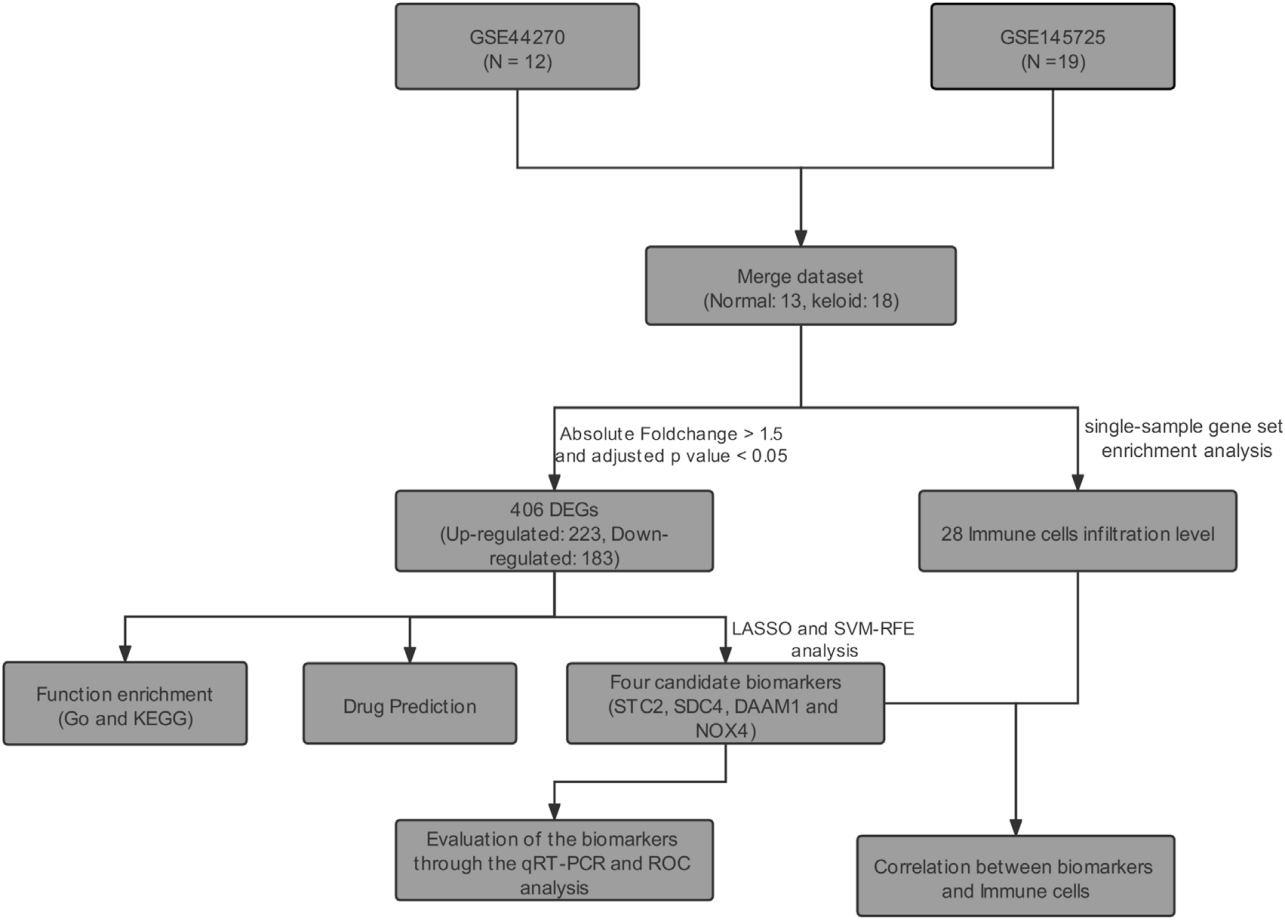
Flowchart of this study’s analysis protocol.

We downloaded all the original expression profile data of GSE44270 (Hahn et al., 2013) and GSE145725 (Kang et al., 2020) from the GEO database of NCBI. The GSE44270 dataset contained 3 keloid lesion samples and 9 normal skin samples. The GSE145725 dataset contained 9 keloid lesion samples and 10 normal skin samples. The details of GSE44270 dataset are shown in Supplementary Table S1. However, the demographic information for GSE145725 dataset had not been uploaded on NCBI GEO Datasets. Two datasets of original data were firstly visualized using box plots, and the background corrected using the sva R package (Leek et al., 2012). Then, we adjusted abnormally distributed data. Batch effect of both datasets was removed using sva R package, and the distributions of samples before and after correction were evaluated using principal component analysis (PCA). The downstream analysis is visualized in Figure 1.

### Identification of Differentially Expressed Genes

DEGs between normal fibroblasts (NFs) and normalized Keloid fibroblasts (KFs) groups were screened using limma R package (Ritchie et al., 2015), according to adjusted *p*-value <0.05 and fold-change >1.5.

### Enrichment Analysis

GO and Disease Ontology (DO) analyses were conducted using clusterProfiler package, respectively (Yu et al., 2012). The enriched functions and locations of genes were described. In addition, difference in biological performance between KFs group and NFs group was analyzed by clusterProfiler package-based Gene Set Enrichment Analysis (GSEA). The false discovery rate (FDR) was set at <0.05.

### PPI Network Analysis

A PPI network was constructed with the screened DEGs based on Search Tool for the Retrieval of Interacting Genes (STRING) database (http://www.string-db.org/). The confidence level was set as ≥0.9. The core modules were filtered by Cytoscape (3.7.1) plug-in MCODE (Liu et al., 2017). The thresholds were set as follows: freedom degree of 2, the K-Core value of 2 by default, and the node score of 0.2.

### Screening of Biomarkers

In order to further screen the core genes from the PPI network, the Glmnet package was used to perform the minimum absolute value convergence and LASSO for variable selection (Friedman et al., 2010). The CV.GLMNET function in the GLMNET package was used to make model cross-validation analysis. The parameter was set as Family = “binomial”, n_folds = 10. The SVM algorithm was used for the gene screening. In the current study, variables were selected by SVM recursive feature elimination (SVM-RFE) algorithm (Huang et al., 2014). The thresholds were set as follows: k = 5, halve.above = 100. Results of Lasso and SVM analyses were further analyzed to determine the key genes.

### Validation of Potential Biomarkers

The genes identified in LASSO and SVM-RFE analyses were further evaluated by the receive operator curve (ROC) analysis for their specificity and sensitivity. The expression levels of Stanniocalcin-2 (STC2), Syndecan-4 (SDC4), NADPH oxidase 4 (NOX4), and Dishevelled Associated Activator Of Morphogenesis 1 (DAAM1) were measured using real-time qPCR (qRT-PCR). Later, we collected keloid lesions and matched healthy skin samples from 8 KD patients for qRT-PCR detection. The experiment gained approval from the Institutional Ethics Committee of the Chinese Academy of Medical Science and Peking Union Medical College (Ethical Approval Number: 2017-ky-006). Patients signed written consent. The primary fibroblasts were extracted from the tissue of both groups, and subjected to following experiments. TRIzol (Thermo Fisher Scientific, United States) was adopted for extracting total RNA. Meanwhile, the NanoDrop 2000 (Thermo Fisher Scientific, United States) was utilized to measure RNA purity and content. Subsequently, RNA was reverse-transcribed to provide cDNA. The qPCR reaction system contained SYBR Green Master Mix, cDNA template and primers. The StepOnePlus Real-Time PCR machine (Thermo Fisher, United States) was employed for real-time qPCR at 95°C for 15 s, 60°C for 60 s, 40 cycles in total. The primers used for amplification are specified in Supplementary Table S2.

### Evaluation of ICI

This study adopted the single-sample GSEA (ssGSEA) algorithm to quantify relative abundances of 28 immune cells from all samples from the GEO dataset. We acquired all gene sets about immune cells in the previous study (Charoentong et al., 2017). The enrichment score for each sample was estimated as the ICI level in KFs samples.

### Analysis of Correlation Between Biomarkers and ICI

Spearman correlation was carried out to analyze the associations of hub genes with 28 kinds of infiltrating immune cells by “ggstatsplot” package (https://github.com/IndrajeetPatil/ggstatsplot). The results were visualized using “ggplot2” package.

### Identification of Candidate Small Molecule Drugs

In the Connectivity Map Database (CMAP), the gene expression signatures were utilized for predicting low-molecular-weight compounds in a certain disorder (https://portals.broadinstitute.org/cmap/). In this work, we classified DEGs into up-regulated or down-regulated groups, and imported them into CMAP to identify small-molecular-weight drugs for KD treatment. The correlation between drugs and DEGs was marked with scores ranging −1 to 1. The negative score indicated that the DEGs might be adopted for KD treatment. We selected potential drugs to treat KD at the threshold of ≤ −0.50.

### Statistical Analysis

The statistical analysis was conducted on SPSS software (version 19.0) and R software (version 4.0.2). Continuous data in normal distribution were expressed as mean ± SD. Paired t test was adopted to compare gene expression levels in KF and NF groups. For all the statistical tests involved in this work, *p* < 0.05 indicated statistical significance.

## Results

### Data and Batch Effect

**FIGURE 2:**
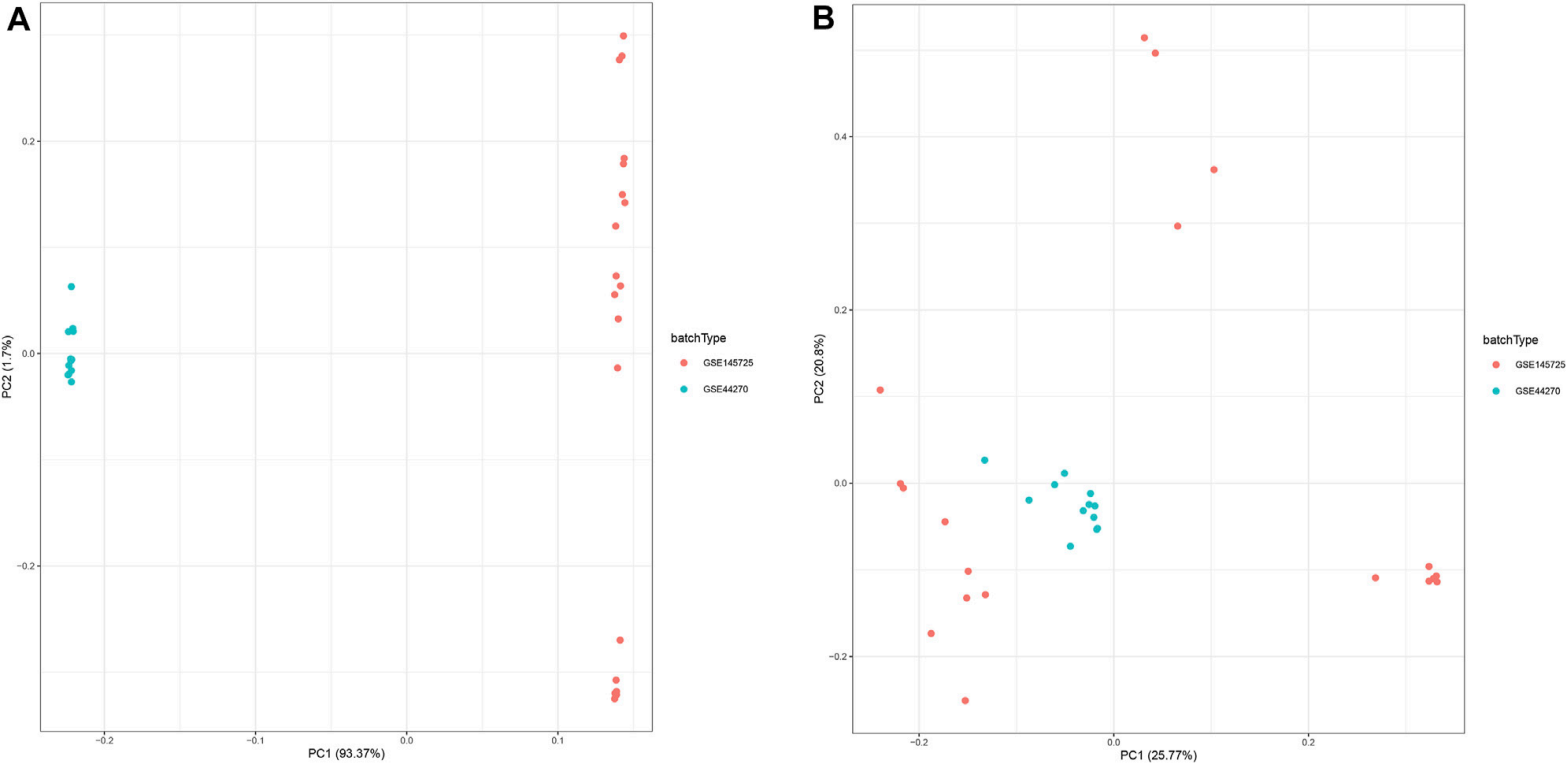
Principal component analysis (PCA) of the gene expression datasets. The points of the scatter plots represented each samples based on the top two principal components (PC1 and PC2) of gene expression profiles without **(A)** and with **(B)** the removal of batch effect.

In this study, we collected two datasets (GSE44270 and GSE145735) of keloid from the GEO database. Among them, GSE44270 was performed on GPL6244 though Affymetrix human gene 1.0 st microarray and was composed to 3 keloid lesion samples and 9 normal skin samples; GSE145725 was conducted on GPL16043 through GeneChip®PrimeView™ human gene expression microarray and containing 9 keloid lesion samples and 10 normal skin samples. Considering the sample size of the keloid sample was small, we thus integrated the two dataset after removed the batch effect. As a result, a total of 15,807 genes were detected in the merge dataset, and the principal component analysis result indicated that the batch effect had been successfully eliminated (Figure 2).

### Identification of DEGs

**FIGURE 3:**
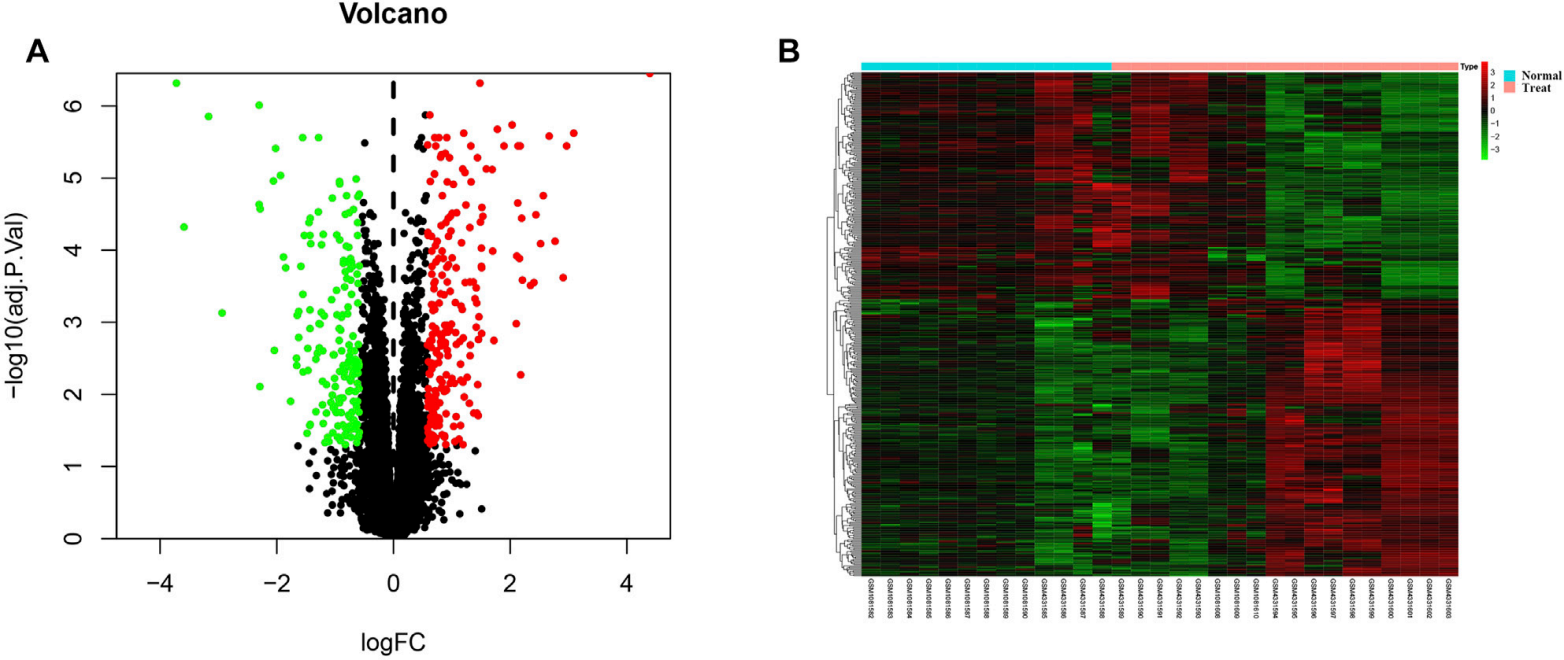
Identification of differentially expressed genes. **(A)** Volcano plot for all genes, the green dot represent the down-regulated genes, red dot indicated the up-regulated genes, while the black genes showed the non-significant genes. **(B)** The differentially expression genes were plotted by a heatmap, the bright red bar indicated the higher gene expression level, while the bright green indicated the lower gene expression level.

The DEGs between keloid and normal tissue were identified by using the limma R package, and a total of 406 DEGs, including 223 up-regulated and 183 down-regulated were screened. Figure 3A showed the distribution of the DEGs, the blue dot represents the down-regulated genes, while the red dot represent the up-regulated genes. In addition, all DEGS were further visualized in the heatmap and showed a tissue-specific expression (Figure 3B).

### Enrichment Analysis

**FIGURE 4:**
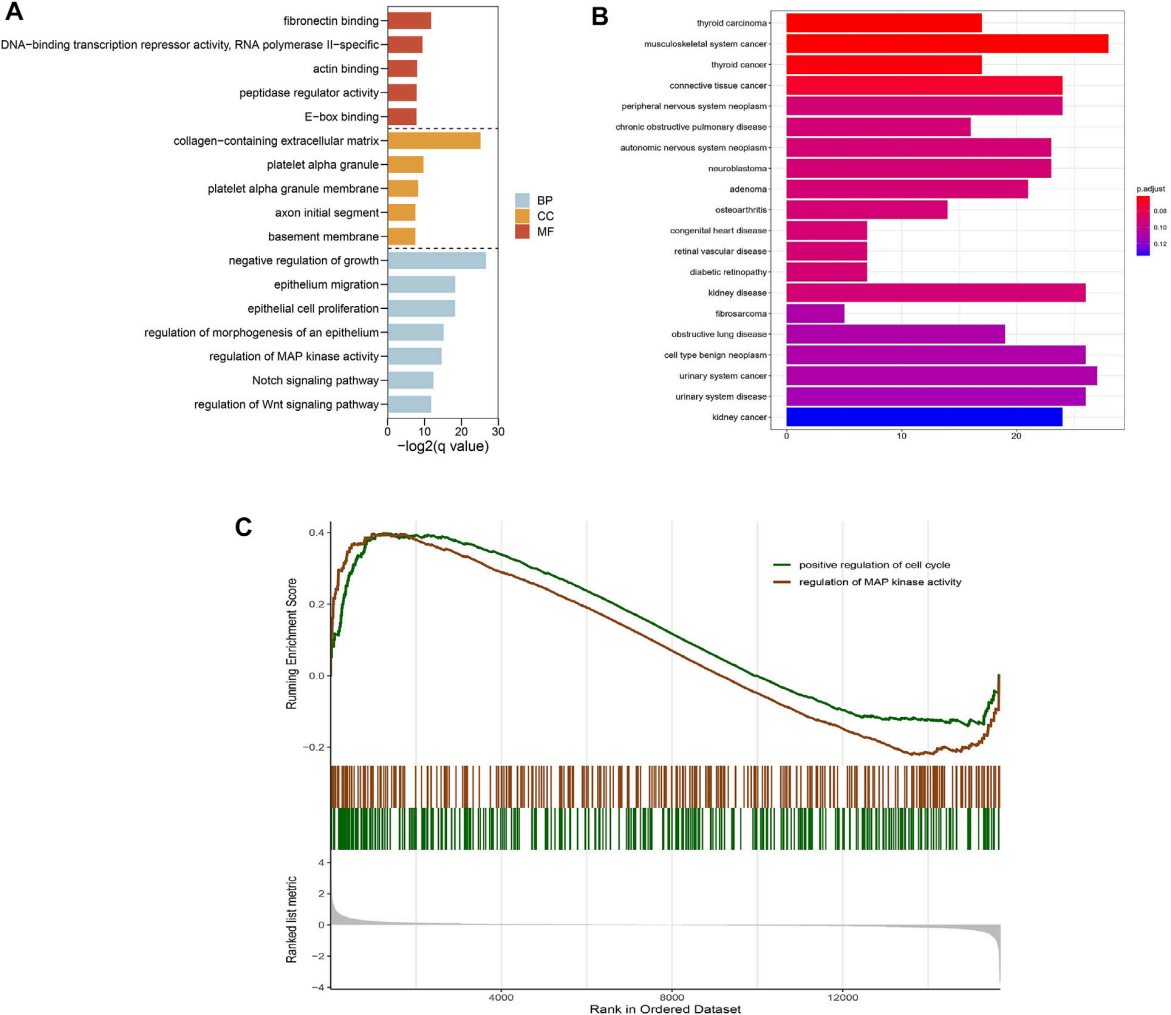
GO enrichment of DEGs and GSEA enrichment analysis for all gene expression matrix. **(A)** GO enrichment analysis, where the horizontal axis represents the number of DEGs under the GO term. **(B) **DO enrichment analysis, where the horizontal axis represents the number of DEGs under the DO term.**(C)** Two pathways including positive regulation of cell cycle and regulation of MAP kinase activity were significantly enriched by the GSEA analysis.

To explore the molecular function and potential pathways of these DEGs, we further carried out the GO and KEGG analysis. The GO analysis result revealed the DEGs involved in negative regulation of growth, epithelium migration, regulation of MAP kinase activity, Notch signaling pathway and regulation of Wnt signaling pathway (Figure 4A). DO analysis results showed that the DEGs were significantly enriched in musculoskeletal system cancer, thyroid carcinoma and connective tissue cancer (Figure 4B). In addition, the GSEA enrichment analysis showed that positive regulation of cell cycle and regulation of MAP kinase activity were enriched in keloid (Figure 4C).

### PPI Network and Key Modules

**FIGURE 5:**
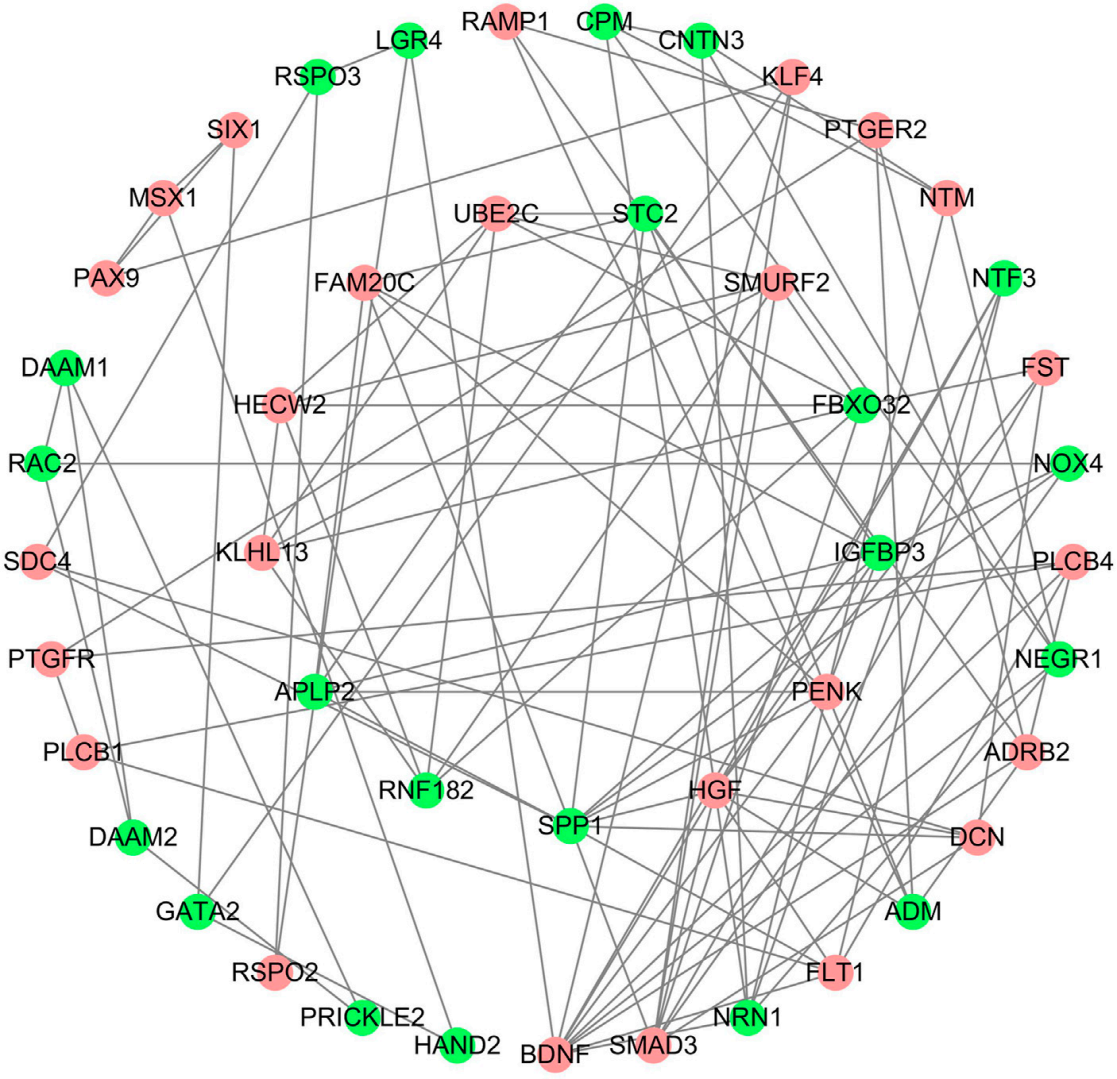
PPI network construction, critical module from PPI network. Green circles: down-regulation with a fold change of more than 2; red circles: upregulation with fold change of more than 2.

We further constructed a protein-protein interaction (PPI) network of DEGs through cytoscape software. A total of 349 nodes and 988 edges were identified according to STRING database-derived data (Supplementary Figure S1). The key sub network was further analyzed using the MCODE algorithm and the top two hub networks were acquired (Figure 5).

### Screening of Biomarkers

**FIGURE 6:**
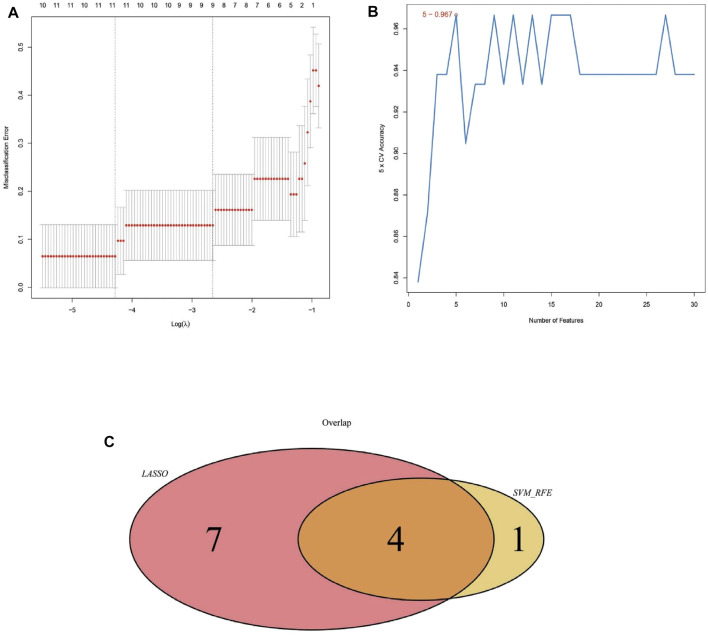
Identification of key genes through LASSO and SVM analysis. **(A)** Cross-validation for tuning parameter selection in the LASSO regression model, dotted vertical lines were drawn at the optimal values, and 11 genes were screened. **(B)** SVM-RFE algorithm was applied to screened genes. The red dots indicated the number of genes achieving the highest accuracy. **(C)** The venn diagram showed the intersection of the genes that obtained from the LASSO and SVM-RFE algorithms.

After identified the hub networks from the MCODE analysis result, we further using the two algorithms (LASSO, SVM-RFE) to screen key genes that associated keloid tissue. The LASSO algorithm was used to calculate the best λ value through a 10-fold cross-validation analysis (Figure 6A). The left and right vertical lines represented the minimal CV error curve, and the most formal model with a CV error lower than 1 SD, respectively. Thereafter, lambda.min value was extracted to screen out 11 key genes, including HECT, C2, and WW Domain Containing E3 Ubiquitin Protein Ligase 2 (HECW2), STC2, Follistatin (FST), SMAD Family Member3 (SMAD3), SDC4, Leucine Rich Repeat Containing G Protein-Coupled Receptor 4 (LGR4), DAAM1, Decorin (DCN), Phospholipase C Beta 1 (PLCB1), Paired Box 9 (PAX9), NOX4. Then, the SVM-RFE algorithm was used to screen five key genes including DAAM1, SDC4, PTGER2, STC2, and NOX4 (Figure 6B). Finally, we used the Venn diagram (Figure 6C) to intersect the key genes screened through the two algorithms, and 4 key genes, including STC2, SDC4, DAAM1, and NOX4 were screened from the two algorithms.

### Verification of Biomarkers

**FIGURE 7:**
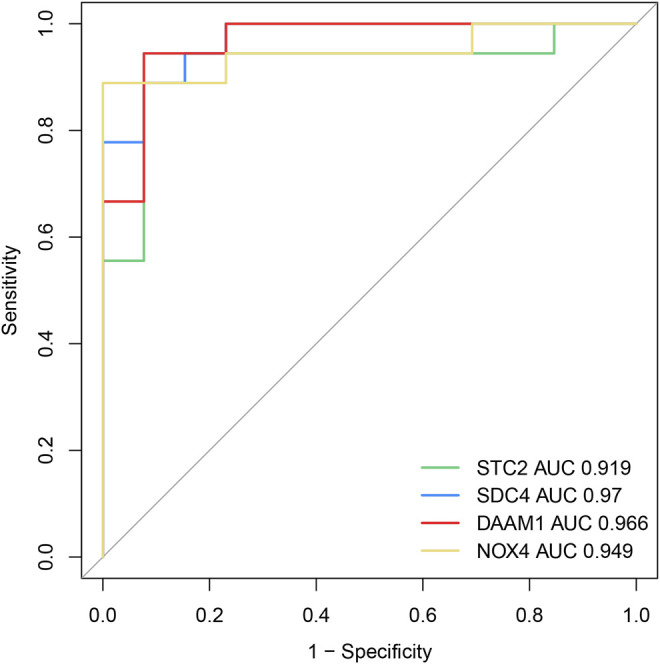
The diagnostic efficacy of the four diagnostic markers were evaluated by the ROC curve analysis.

**FIGURE 8:**
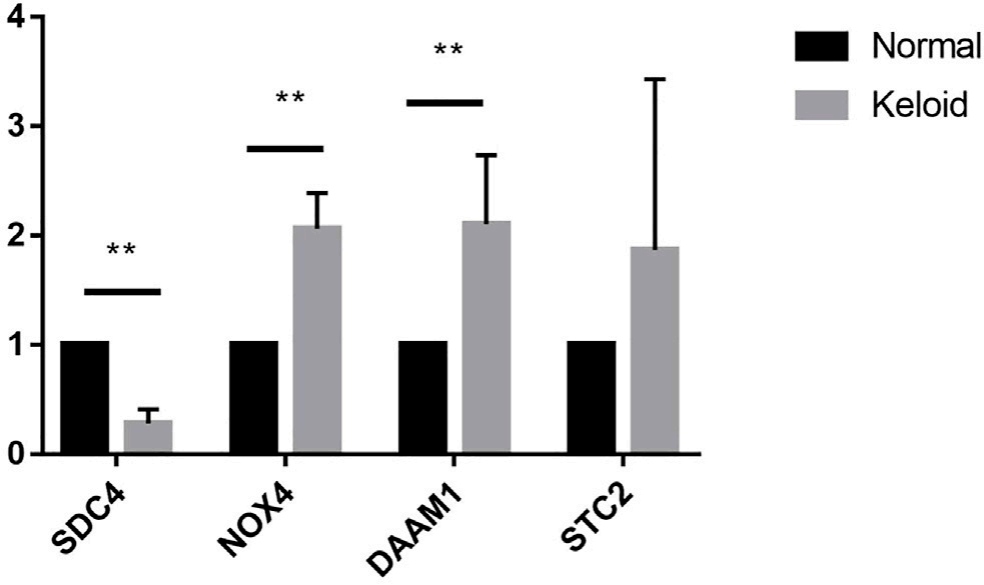
The expression differences of SDC4, NOX4, DAAM1, STC2 between keloid fibroblasts and normal skin fibroblasts. (Note: **compared the keloid tissues with the normal human skin tissues. *p* < 0.01)

To investigate the diagnostic value of the four genes, we then conducting a ROC analysis. The areas under the curve (AUCs) value of STC2, SDC4, NOX4, and DAAM1 were 0.919, 0.970, 0.949, and 0.966, respectively, suggesting that the four genes had a high diagnostic value (Figure 7). In addition, we also conducting a qRT-PCR experiment for detecting differential expression of SDC4, NOX4, DAMM1, and STC2 between keloid and normal tissue. Compared to normal group, the relative expression levels of SDC4, NOX4, DAMM1, and STC2 in keloid fibroblasts were 0.28 ± 0.13, 2.07 ± 0.32, 2.11 ± 0.63, and 1.87 ± 1.56, respectively (Figure 8). The expression levels of SDC4, NOX4, and DAMM1 showed a significantly statistical differences between groups (*p* < 0.05).

### Immune Cell Infiltration Results

**FIGURE 9:**
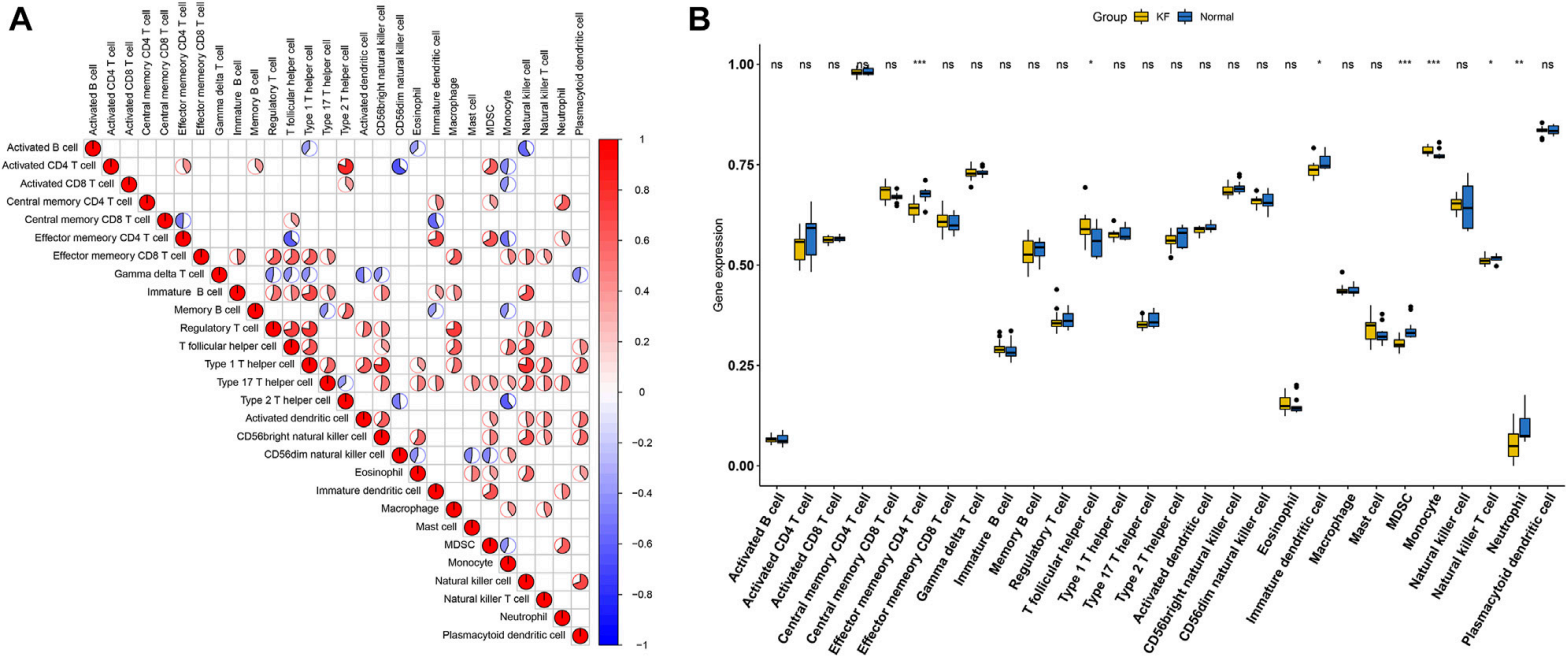
Evaluation and visualization of immune cell infiltration. **(A)** Correlation heat map of 28 types of immune cells. The size of the colored circless represents the strength of the correlation; red represents a positive correlation, blue represents a negative correlation. The darker the color, the stronger the correlation. **(B)** The diagram of the difference in infiltration between the two groups of samples of proportion of 28 types of immune cells. (Note: ns Compared with control group, *p* > 0.05; * Compared with control group, *p* < 0.05; **compared the keloid tissues with the normal human skin tissues. *p* < 0.01; ***compared the keloid tissues with the normal human skin tissues. *p* < 0.001)

Considering the association between immune cell and keloid were unclear, we further explored the immune cell infiltration of the keloid tissue through the ssGSEA algorithm. As showed in Figure 9A, we observed that the activated CD4 T cells showed a significant positive correlation with type 2 T helper cells, while Type 1 T helper cells and T follicular helper cells were in direct proportion to regulatory T cells. In addition, we also compared the immune cell infiltration between keloid tissue and normal tissue. We discovered that the infiltration level of T follicular helper cell and Monocyte were significantly higher in the keloid tissue, while Effector memeory CD4 T cell, Immature dendritic cell, MDSC, Natural killer T cell and Neutrophil presented a high level in normal tissue (Figure 9B).

### Correlation Between Biomarkers and Infiltrating Immune Cells

**FIGURE 10:**
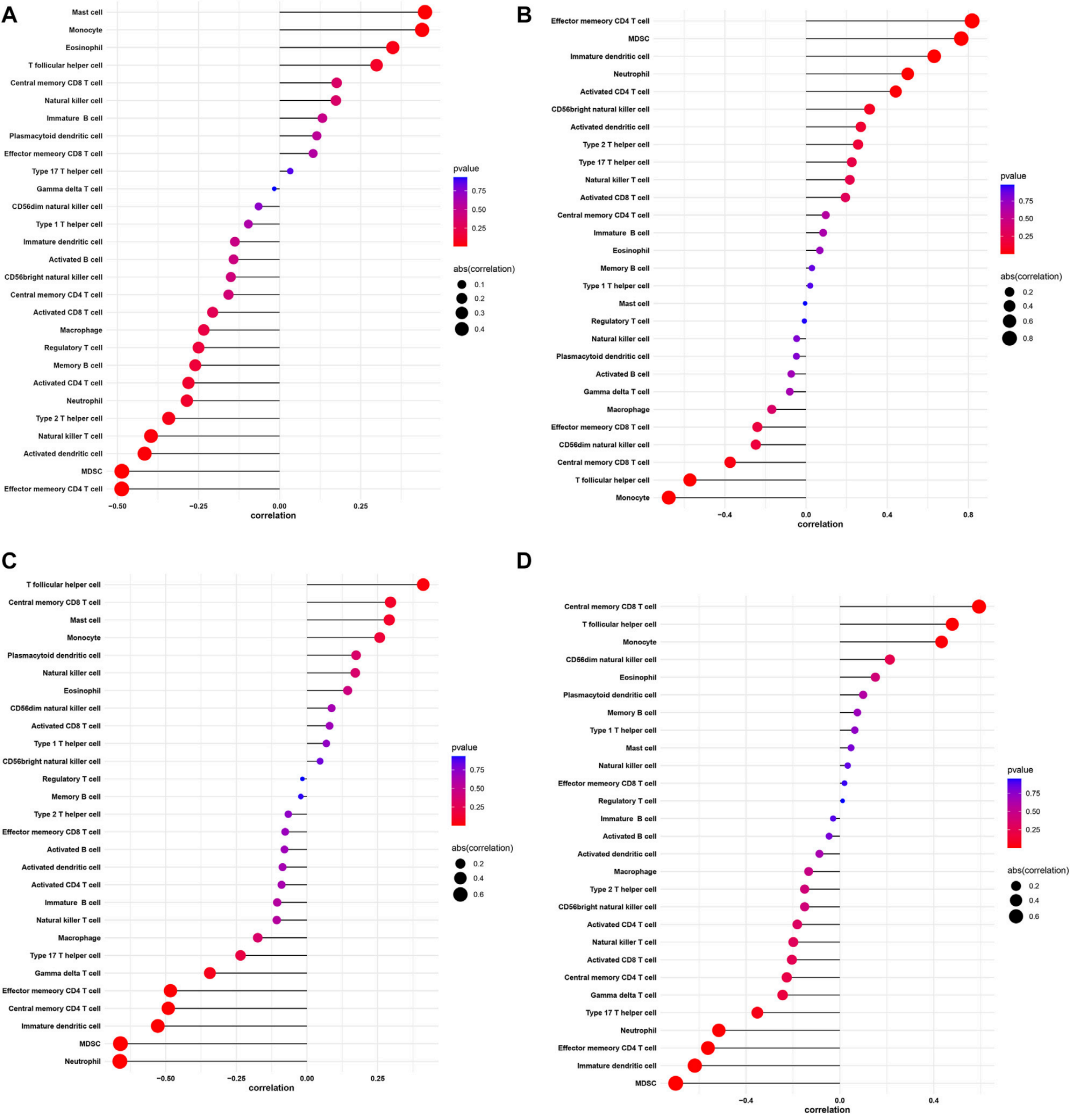
Correlation between DAAM1, SDC4, STC2, NOX4 and infiltrating immune cells. **(A)** Correlation between DAAM1 and infiltrating immune cells. **(B)** Correlation between SDC4 and infiltrating immune cells. **(C)** Correlation between STC2 and infiltrating immune cells. **(D)** Correlation between NOX4 and infiltrating immune cells. The size of the dots represents the strength of the correlation between genes and immune cells; the larger the dots, the stronger the correlation, and the smaller the dots, the weaker the correlation. The color of the dots represents the *p*-value, the redder the color, the lower the *p*-value, and the bluer the color, the larger the *p*-value.

Using the ssGSEA algorithm to calculate the enrich score of 28 immune cells in all samples from GEO database, we explored the relationship between 28 immune cells and four key genes through spearman correlation analysis on keloid tissue. We found that the DAAM1 showed a positive correlation with mast cells and monocytes, and a negative correlation with effector memory CD4 T cells and MDSCs (Figure 10A). In addition, SDC4 showed a positive correlation with effector memory CD4 T cells, but a negative correlation with monocytes (Figure 10B). STC2 was positively correlated with T follicular helper cells and negatively correlated with MDSCs and neutrophils (Figure 10C). NOX4 was in direct proportion to central memory CD8 T cells, but indirect proportion to MDSCs (Figure 10D).

### Prediction of Candidate Drugs

To identify the candidate drugs for the treatment of keloid tissue, all DEGs were imported into CMAP drug database. A negative value of the drugs might have power to reverse the clinical manifestations in the keloid patients. As a result, 12 drugs including mercaptopurine, melatonin, sulmazole, parthenolide, chloropyrazine, pheneticillin, iopamidol, estriol, chlorogenic acid, CP-863187, GW-8510, isoxicam, dilazep were screened. Notably, mercaptopurine with an enrichment score of −0.923 showed extraordinary therapeutic potential (Supplementary Table S3).

## Discussion

The pathophysiological mechanisms of keloid formation remain unclear (Lv et al., 2020). Due to the lack of useful targets for anti-keloid therapies, keloid frequently recurs, compromising the quality of life of many patients. Therefore, finding keloid-related biomarkers and exploring the effect of ICI on KD are of profound significance.

In this present study, innovatively combining SVM-RFE and LASSO-logistic regression methods, we identified four biomarkers (STC2, SDC4, DAAM1, and NOX4) for keloid and further explored the role of ICI on keloid. Then we conducted a ROC analysis to investigate the diagnostic value of the four genes. Finally, we validated the expression of genes in eight keloid samples.

Stanniocalcin (STC) is a glycoprotein hormone found within the Stannius corpuscles of bony fish (Flik et al., 1990). STC2, a member of the STC family, is a kind of glycoprotein involved in the homeostatic regulation of calcium and phosphorus. Previous studies have shown that STC2 is overexpressed in several cancers with poor prognosis (Law and Wong, 2010). In ovarian cancer, overexpressed STC2 promotes epithelial-mesenchymal transition, as manifested by the increase in N-cadherin/vimentin loose fibroblastic colonies and the mesenchymal marker expression, and the decrease in epithelial marker expression (Law and Wong, 2010). In addition, cadherin expression is lowly and vimentin is highly expressed in KD (Yan et al., 2015).

NADPH oxidase 4(NOX 4) is a dual heme-containing enzyme that spans the membrane 6 times and produces reactive oxygen species, namely hydrogen peroxide (Gray et al., 2019). NOX4 is mainly related to cell growth, apoptosis, differentiation, ECM synthesis and secretion. In lung fibroblasts (Amara et al., 2010) and kidney fibroblasts (Bondi et al., 2010), NOX4 can act as a Smad2/3 activator to regulate collagen expression mediated by TGF-β. In an *in-vitro* study of lung fibroblasts collected from cases with idiopathic pulmonary fibrosis (IPF), Nadia Amara and others Amara et al. (2010) discovered the positive correlation between NOX4 expression and the levels of α-smooth muscle actin as well as type I collagen. Therefore, NOX4 possibly plays an important part in ECM production and deposition within KD, and the formation of abnormal collagen bundles.

Syndecan-4(SDC4), a universally expressed transmembrane proteoglycan bearing heparan sulfate chains, is involved in numerous inside-out and outside-in signaling processes (Keller-Pinter et al., 2021). SDC4 regulates cell migration, proliferation, differentiation, adhesion and apoptosis in inflammation and wound healing (Frey et al., 2013). Previous *in-vitro* studies on lung fibroblasts have shown that SDC4 can regulate TGF-β signal transduction by separating activated TGF-β from its β receptor; then with the inactivation of Smad3, the collagen and α-smooth muscle actin are up-regulated to further modulate ECM expression (Tanino et al., 2019). These studies show that syndecan-4 can inhibit pulmonary fibrosis (PF) progression through repressing TGF-β signaling.

Dishevelled associated activator of morphogenesis 1 (DAAM1), a member of formin protein family that functions as central players in cytoskeletal reorganization, participates in the WNT/planar cell polarity (PCP) pathway (Sato et al., 2006; LaMonica et al., 2009). Studies have found that reducing the protein level of DAAM1 can efficiently suppress tumor cell colony formation, invasion, and migration (LaMonica et al., 2009). However, studies on DAAM1 are scarce, and further exploration is still needed.

In this study, we examined the infiltration level of 28 immune cells in the samples from GEO database. We found that T follicular helper cells, effector memory CD4 T cells, MDSCs, NK T cells, immature DCs, and neutrophils exhibited significant differences between keloid and the normal tissue. Previous studies have shown a higher level of memory T cell in keloid tissue than in healthy skin, with altered cellular composition (Chen et al., 2018). CD8^+^ memory T cell proportion increases in keloid tissue relative to that in normal skin tissue, which is usually detected in inflammatory skin disease (Chen et al., 2018). Researchers have verified distinct Th2 signature within KD, together with the higher infiltration levels of immune cells (Zhang et al., 2009; Lee et al., 2020; Wu et al., 2020). However, the role of T follicular helper cells in KD has not been elucidated. Excess immature DCs accumulate in the case of human fibrotic interstitial pulmonary lesion (Marchal-Sommé et al., 2007; Freynet et al., 2016). Recent research has suggested that neutrophils under inflammatory stimulation produce extracellular DNA, a mechanism known as neutrophil extracellular traps that can enhance fibroblast differentiation to promote fibrosis (Chrysanthopoulou et al., 2014). Based on our findings and the above-mentioned studies, immature DCs, memory T cells, neutrophils and T helper cells exhibit critical functions in keloid tissue. Additionally, this study illustrated the correlations between potential biomarkers and immune cells. We found that DAAM1, SDC4, STC2, and NOX4 interacted with immune cells. This interaction should be illustrated with more studies. We also retrieved small molecule drugs in the CMAP database, finding that the top-ranked drugs, such as mercaptopurine and melatonin, may have therapeutic potential for KD.

Certain limitations should be noted in the present work. First, the number of patients was still relatively small, which may amplify individual differences. Second, the creditability of our results should be further verified in experiments. Third, our study was inspired by findings in tumor research, but keloid have not been proven to be a real tumor, so more experiments were are needed to make our findings more persuasive.

## Conclusion

In conclusion, we identified four genes that can be served as an potential biomarkers for the keloid and revealed the landscape of immune infiltration in KD, which might provide new insight into the pathogenesis and treatment of KD.

## Data Availability

The original contributions presented in the study are included in the article/Supplementary Material, further inquiries can be directed to the corresponding author.

## Supplementary Material

Supplementary Figure S1 Protein-protein interaction network of differentially expressed genes.
