# Supplementary material for: Keloid Biomarkers and Their Correlation With Immune Infiltration
Source: Front Genet. 2022 Jun 2;13:784073. doi: 10.3389/fgene.2022.784073 (PMC9201286; doi:10.3389/fgene.2022.784073)
Supplement: Supplementary file 1 [file Table3.DOC]

**Supplementary Table3**.The information of potential small molecule drugs predicted by CMAP database for treatment of keloid.

| CMAP name | enrichment | ***p*** |
| --- | --- | --- |
| mercaptopurine | -0.923 | 0.01213 |
| melatonin | -0.863 | 0.00064 |
| sulmazole | -0.825 | 0.01076 |
| parthenolide | -0.766 | 0.00607 |
| chloropyrazine | -0.759 | 0.00684 |
| pheneticillin | -0.759 | 0.00688 |
| iopamidol | -0.744 | 0.00857 |
| estriol | -0.696 | 0.01796 |
| chlorogenic acid | -0.692 | 0.01922 |
| CP-863187 | -0.684 | 0.02186 |
| GW-8510 | -0.665 | 0.02851 |
| isoxicam | -0.661 | 0.01077 |
| dilazep | -0.66 | 0.01087 |
