# Supplementary figures and images for: Keloid Biomarkers and Their Correlation With Immune Infiltration
Source: Front Genet. 2022 Jun 2;13:784073. doi: 10.3389/fgene.2022.784073 (PMC9201286; doi:10.3389/fgene.2022.784073)

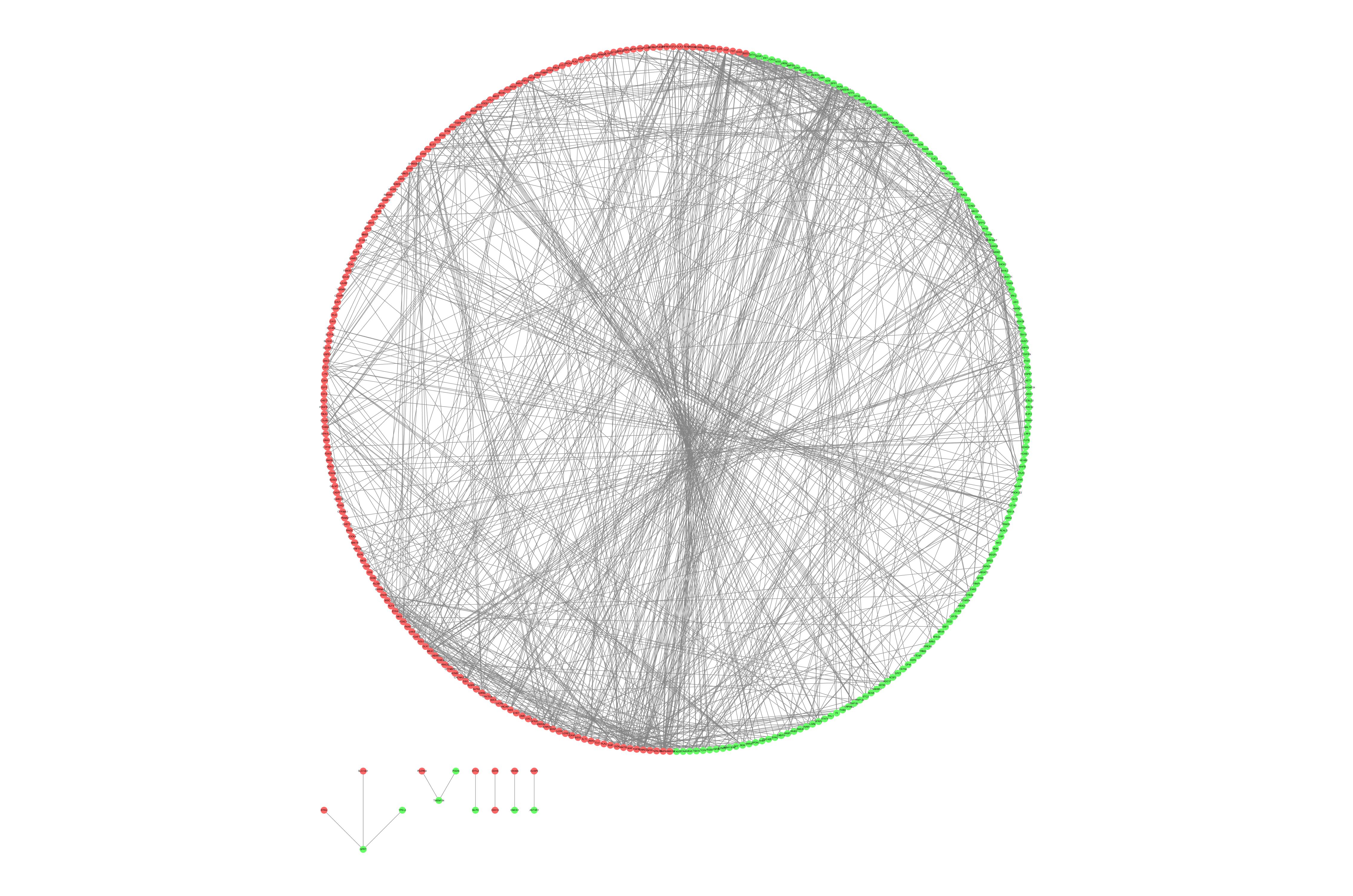

Supplement: Supplementary file 2 [file Image1.JPEG]
