# Supplementary material for: Keloid Biomarkers and Their Correlation With Immune Infiltration
Source: Front Genet. 2022 Jun 2;13:784073. doi: 10.3389/fgene.2022.784073 (PMC9201286; doi:10.3389/fgene.2022.784073)
Supplement: Supplementary file 3 [file Table1.DOC]

Supplementary Table1. The pathological characteristics of GSE44270

| ID | Group | Gender | Ethnicity | Age |
| --- | --- | --- | --- | --- |
| GSM1081582 | Keloid fibroblast | Male | Caucasian | 10 |
| GSM1081583 | Keloid fibroblast | Male | Caucasian | 17 |
| GSM1081584 | Keloid fibroblast | Male | Caucasian | 8 |
| GSM1081585 | Keloid fibroblast | Male | Caucasian | 11 |
| GSM1081586 | Keloid fibroblast | Male | African American | 10 |
| GSM1081587 | Keloid fibroblast | Male | African American | 10 |
| GSM1081588 | Keloid fibroblast | Female | African American | 20 |
| GSM1081589 | Keloid fibroblast | Female | African American | 17 |
| GSM1081590 | Keloid fibroblast | Male | African American | 15 |
| GSM1081608 | Control fibroblast | Female | African American | 16 |
| GSM1081609 | Control fibroblast | Male | Caucasian | 23 |
| GSM1081610 | Control fibroblast | Female | African American | 17 |
