# Supplementary material for: Keloid Biomarkers and Their Correlation With Immune Infiltration
Source: Front Genet. 2022 Jun 2;13:784073. doi: 10.3389/fgene.2022.784073 (PMC9201286; doi:10.3389/fgene.2022.784073)
Supplement: Supplementary file 4 [file Table2.DOC]

**Supplementary Table2**.Primer sequence of qRT-PCR

|  | Primer | Sequence(5'- 3') |
| --- | --- | --- |
| DAMM1 | The forward primer | ATGGCCAAGGCTGATAGGTT |
|  | The reverse primer | CTTGAGGGCACCACTCCTAA |
| NOX4 | The forward primer | TGCTGACGTTGCATGTTTCA |
|  | The reverse primer | TTCTGAGAGCTGGTTCGGTT |
| SDC4 | The forward primer | ACTTTGAGCTGTCTGGCTCT |
|  | The reverse primer | CCTGCCCTCTCAGGGATATG |
| STC2 | The forward primer | TGTCTGCAAAGGGCCAGATA |
|  | The reverse primer | TCAGCTGGGAGCCTGTTAAA |
